# Multimorbidity patterns in COVID-19 patients and their relationship with infection severity: MRisk-COVID study

**DOI:** 10.1371/journal.pone.0290969

**Published:** 2023-08-31

**Authors:** Marina Lleal, Celia Corral-Vazquez, Montserrat Baré, Ricard Comet, Susana Herranz, Francisco Baigorri, Antonio Gimeno-Miguel, Maria Raurich, Cristina Fortià, Marta Navarro, Beatriz Poblador-Plou, Marisa Baré

**Affiliations:** 1 Institutional Committee for the Improvement of Clinical Practice Adequacy, Clinical Epidemiology and Cancer Screening Department, Parc Taulí Hospital Universitari, Institut d’Investigació i Innovació Parc Taulí (I3PT-CERCA), Universitat Autònoma de Barcelona, Sabadell, Spain; 2 Department of Paediatrics, Obstetrics and Gynaecology, Preventive Medicine and Public Health, Autonomous University of Barcelona (UAB), Bellaterra, Spain; 3 Research Network on Health Services in Chronic Patients (REDISSEC), Instituto de Salud Carlos III, Madrid, Spain; 4 Creu Alta Primary Care Centre, Institut Català de la Salut, Sabadell, Spain; 5 Acute Geriatric Unit, Centre Sociosanitari Albada, Parc Taulí Hospital Universitari, Institut d’Investigació i Innovació Parc Taulí (I3PT-CERCA), Universitat Autònoma de Barcelona, Sabadell, Spain; 6 Intensive Care Unit, Parc Taulí Hospital Universitari, Institut d’Investigació i Innovació Parc Taulí (I3PT-CERCA), Universitat Autònoma de Barcelona, Sabadell, Spain; 7 EpiChron Research Group, Aragon Health Sciences Institute, IIS Aragón, Miguel Servet University Hospital, Zaragoza, Spain; 8 Health Record / Information Management, Parc Taulí Hospital Universitari, Institut d’Investigació i Innovació Parc Taulí (I3PT-CERCA), Universitat Autònoma de Barcelona, Sabadell, Spain; 9 Infectious Diseases Department, Parc Taulí Hospital Universitari, Institut d’Investigació i Innovació Parc Taulí (I3PT-CERCA), Universitat Autònoma de Barcelona, Sabadell, Spain; 10 Can Rull – Can Llong Primary Care Centre, Parc Taulí Hospital Universitari, Institut d’Investigació i Innovació Parc Taulí (I3PT-CERCA), Universitat Autònoma de Barcelona, Sabadell, Spain; 11 Research Network on Chronicity, Primary Care and Health Promotion (RICAPPS), Instituto de Salud Carlos III, Madrid, Spain; Nnamdi Azikiwe University, NIGERIA

## Abstract

**Background:**

Several chronic conditions have been identified as risk factors for severe COVID-19 infection, yet the implications of multimorbidity need to be explored. The objective of this study was to establish multimorbidity clusters from a cohort of COVID-19 patients and assess their relationship with infection severity/mortality.

**Methods:**

The MRisk-COVID Big Data study included 14 286 COVID-19 patients of the first wave in a Spanish region. The cohort was stratified by age and sex. Multimorbid individuals were subjected to a fuzzy c-means cluster analysis in order to identify multimorbidity clusters within each stratum. Bivariate analyses were performed to assess the relationship between severity/mortality and age, sex, and multimorbidity clusters.

**Results:**

Severe infection was reported in 9.5% (95% CI: 9.0–9.9) of the patients, and death occurred in 3.9% (95% CI: 3.6–4.2). We identified multimorbidity clusters related to severity/mortality in most age groups from 21 to 65 years. In males, the cluster with highest percentage of severity/mortality was *Heart-liver-gastrointestinal* (81–90 years, 34.1% severity, 29.5% mortality). In females, the clusters with the highest percentage of severity/mortality were *Diabetes-cardiovascular* (81–95 years, 22.5% severity) and *Psychogeriatric* (81–95 years, 16.0% mortality).

**Conclusion:**

This study characterized several multimorbidity clusters in COVID-19 patients based on sex and age, some of which were found to be associated with higher rates of infection severity/mortality, particularly in younger individuals. Further research is encouraged to ascertain the role of specific multimorbidity patterns on infection prognosis and identify the most vulnerable morbidity profiles in the community.

**Trial registration:**

NCT04981249. Registered 4 August 2021 (retrospectively registered).

## Background

The first wave of the COVID-19 pandemic caused high rates of death and critical infection. The first studies quickly identified a differential vulnerability depending on age and sex, so that higher rates of death and severity were detected in older and male individuals [1, 2].

The study of the relationship between isolated chronic diseases and COVID-19 infection has resulted in the identification of diverse morbidities as potential risk factors, such as diabetes mellitus (DM), obesity, hypertension, heart failure, chronic obstructive pulmonary disease (COPD), chronic kidney disease, and cardiovascular (CV) conditions [3–10]. Noticeably, many of these conditions share some pathophysiological pathways, suggesting that their interactions could increase the risk of a poor COVID-19 prognosis, and that their identification would help in the management of the disease. Nevertheless, few research works have examined the interaction between multiple diseases and its impact on COVID-19 outcomes [8, 11–13].

Multimorbidity (MM), which is traditionally defined as the concomitance of two or more chronic conditions (CC) in the same individual, is a highly prevalent issue that increases with age and society ageing [14–17]. Multimorbid patients may present complex profiles associated to pathophysiological interactions that could complicate medical treatment [18, 19]. However, there is no gold standard in the characterization of MM due to the novelty of the research field, and many studies analyse the mere presence or absence of MM, the sole number of CC, pre-established scores such as the Charlson Multimorbidity Index, or the aggregation of CC by factor analysis or hard hierarchical-clustering methods [20–22].

Remarkably, new strategies that more accurately reflect the complex, non-random association of CC in a patient-centred point of view are emerging, such as soft (or fuzzy) clustering analysis [23–25]. This technique has already provided relevant results in previous studies, so that several relationships between MM patterns and clinically relevant outcomes such as disability, adverse drug reactions, hospitalization, or mortality have been identified [26–30].

In order to perform a solid profiling of the MM patterns, detailed medical information is needed. In this sense, the use of Big Data provides a quick and exhaustive covering of large datasets. Indeed, the access to public healthcare Big Data facilitated by several state organisms enabled rapid analyses, yielding relevant results, and leading to a better handling of the pandemic [13, 31–33].

Given the previous statements, the aim of the present study, encompassed in the MRisk-COVID Big Data project, was to provide an age- and sex-centred characterization of the MM clusters of an adult COVID-19 cohort and to assess the relationship between these clusters and the severity and mortality of the infection.

## Methods

### Study design and cohort

The MRisk-COVID study is an observational Big Data research project based on a cohort of 14286 COVID-19 patients aged >20 years, residing in a healthcare zone of about 400000 inhabitants in the Northeast region of Spain (Vallès Occidental est). This healthcare zone is situated within the autonomous community of Catalonia, specifically within the province of Barcelona, and encompasses a single reference hospital, Parc Taulí Hospital Universitari. Data were provided by the Agency for Health Quality and Assessment of Catalonia (AQuAS) in the framework of the Public Data Analysis for Health Research and Innovation Program (PADRIS), under an extraordinary call that took place in April 2020 in order to provide healthcare Big Data for COVID-19 research studies.

The cohort of this study is composed by all COVID-19 cases registered between 27^th^ February and 15^th^ June of 2020 by the Catalan Epidemiological Surveillance Emergency Service (SUVEC) as either confirmed cases (identified through positive PCR, ELISA or rapid antibody test), or suspected cases (no positive test result, but exhibited symptoms and were classified as potential cases by physicians). The study did not involve sampling, as it encompasses all individuals who met the inclusion criteria. The International Classification of Diseases, 10^th^ edition, Clinical Modification (ICD-10-CM) codes considered as COVID-19 diagnoses are summarized in S1 Table.

### Ethics statement

The study was approved by the Ethics Committee (CEIm) of the Parc Taulí University Hospital (reference 2021/5067), which waived the requirement of informed consents due to the epidemiological nature of the study and the use of anonymized data provided by PADRIS. This study was conducted according to the principles expressed in the Declaration of Helsinki.

### Big data processing, data linkage and study outcomes

Demographic information and clinical records were compiled, integrated and analysed. Demographic data were obtained from the Shared Clinical History of Catalonia (HC3) and included age and sex. In order to prevent re-identification of the patients, age was provided by PADRIS in quinquennia, and groups with high risk of re-identification were not included (women aged >95 and men aged >90). Clinical records were obtained from any primary healthcare centre and hospital of Catalonia that the individuals attended. The provided records covered from 1923 to 2020 in primary healthcare centres, and from 2016 to 2020 in hospitals. These data encompassed all diagnoses and hospital procedures, including the index hospitalization episode of COVID-19 infection. Information about admission and discharge circumstances was also provided (S1 Fig). Patients’ data were stratified by sex and age, thereby obtaining eight different datasets (groups of 21–45, 46–65, 66–80 and 81–95 years, separated into male and female individuals).

Infection severity was defined as the occurrence of at least one of the following conditions during any of the registered COVID-19 episodes: severe respiratory affection (including insufficiency, failure, or distress); use of respiratory support (including mechanical ventilation or oxygen therapy); septic shock; multiple organ failure (the combination of respiratory failure and any other organ failure); inflammatory response; admission to intensive care unit; and mortality associated with COVID-19 episodes (either registered by epidemiological surveillance, primary care or hospital records). This definition was based on the COVID-19 Treatment Guidelines provided by National Institutes of Health [34], with some modifications under the research group physicians’ criteria. The ICD-10-CM and ICD-10 Procedure Coding System (PCS) codes considered as severity conditions are compiled in S1 Table. Besides, mortality was also assessed independently.

Data compilation, processing and statistical analysis were performed using R [35] and RStudio [36].

### Multimorbidity cluster analysis

The ICD-10-CM codes of the complete diagnoses records were categorized into chronic or acute diseases by the Chronic Condition Indicator v.2021.1 [37], and only CC were selected. Then, in order to reduce the number of variables for the cluster analysis, diagnoses were grouped and classified by the Clinical Classification Software Refined v.2021.1 [37]. This software provides a hierarchical classification of the diagnoses into general CC categories (e.g. *Asthma*, or *Chronic obstructive pulmonary disease and bronchiectasis*), which, in turn, belong to disease families (e.g. *Diseases of the respiratory system*). The CC of the family *Neoplasms* were manually distributed into two general categories: *Neoplasia (solid tumour)*, and *Hematologic neoplasia*.

The clustering procedure was independently applied to each sex-age group. For each stratum, CC were filtered by prevalence (>2%) and only patients with MM, as defined by the presence of 2 or more CC, were included in the analyses. The clustering methodology was adapted from the one described by Vetrano et al [28]. An initial dimension reduction of the CC datasets was performed by a Multiple Correspondence Analysis, using the elbow criteria in the Scree plot for dimension selection. The resulting data were subjected to soft clustering analysis by the fuzzy c-means algorithm. This algorithm requires two main parameters: the number of desired clusters (*C*) and the fuzziness (*m*), which indicates the degree of overlapping membership of the patients to the clusters. This parameter can range from 1 (equivalent to hard, non-overlapping clustering) to infinite. Several values of *C* (4, 5, 6 and 7) and *m* (1.1, 1.2, 1.4, 1.5, 2, and 4) were tested. Since fuzzy c-means is a stochastic method, 100 iterations were performed for each combination of *C* and *m* in order to obtain mean and reproducible results. The optimal *m* was estimated by the mean calculation of five indexes: Calinski–Harabasz, Partition coefficient (both optimal at their highest value), Partition entropy, Fukuyama, and Xie-Beni (the three of them optimal at their lowest value).

The final *C* for each sex and age stratum was determined through consensus among 11 professionals of the multidisciplinary research group. This agreement was achieved following a Delphi-like selection method that consisted on equal voting through two selection rounds. Consensus was defined when one of the options reached a majority of votes (≥6); cases with lack of consensus or similar voting count were decided through a clinical debate session.

Several indicators were calculated for the CC in each cluster in order to characterize the specificity and composition of the MM patterns: a) Prevalence within the cluster (%); b) Observed/Expected (O/E) ratio, estimated by dividing the prevalence in the cluster by the prevalence in the corresponding age-sex group cohort; and c) Exclusivity (%), obtained by dividing the number of patients that presented the CC in the cluster by the total number of patients affected by the CC in the corresponding age-sex group cohort. A generic label was assigned to each cluster in an attempt to summarize the most prevalent and overexpressed CC as well as to facilitate the clinical interpretation of the results. Regarding patients, membership percentages were calculated, indicating the degree of belonging of each patient to the selected clusters.

### Statistical analysis

Statistical bivariate differences between severe and non-severe cases, as well as dead and survivor cases, were measured by chi-square tests in the case of categorical/dichotomous variables (age, sex, and MM) and Wilcoxon signed-rank tests in the case of continuous variables (number of CC).

The statistical relationship between mortality / severity and the MM clusters was assessed by weighted chi-square tests, where the weight of the variables was the percentage of membership of the patients to each cluster. Patients with only one or no CC were evaluated as an additional “No multimorbidity” group. These groups were discarded from the analysis of the 66–80 and 81–95 years strata, due to the scarce number of non-multimorbid cases.

## Results

### Characteristics of the study population, infection severity and mortality

The study cohort comprised a total of 5411 males (37.9%) and 8875 females (62.1%). The age distribution revealed a mode of 41–45 years (n = 1438). Among the total cohort, 3140 patients had a confirmed COVID-19 infection (22.0%) and 11 146 were suspected cases (78.0%). The 78.9% of the cohort presented MM, and the mean number of CC was 6.0 (standard deviation of 5.4) (Table 1).

**Table 1:** Demographic and clinical distribution of the study cohort in relation to severe COVID-19 infection/mortality. SD = standard deviation; OR = Odds Ratio; CI = Confidence Interval.

Variable		Total	Severe infection	p-value	OR [95% CI]	Mortality	p-value	OR [95% CI]
Sex. N (%)	Female	8875	621 (7.0)	<0.001	Reference	267 (3.0)	<0.001	Reference
	Male	5411	731 (13.5)		2.1 [1.8–2.3]	288 (5.3)		1.8 [1.5–2.1]
Age. N (%)	21–45	5599	117 (2.1)	<0.001	Reference	9 (0.2)	<0.001	Reference
	46–65	4699	390 (8.3)		4.2 [3.4–5.2]	67 (1.4)		8.8 [4.6–19.2]
	66–80	2299	480 (20.9)		12.3 [10.0–15.3]	210 (9.1)		61.3 [33.4–129.4]
	81–95	1689	365 (21.6)		12.9 [10.4–16.1]	269 (15.9)		115.6 [63.0–245.5]
Multimorbidity. N (%)	No	3014	50 (1.7)	<0.001	Reference	2 (0.1)	<0.001	Reference
	Yes	11 272	1302 (11.5)		7.7 [5.9–10.4]	553 (4.9)		72.1 [23.4–461.7]
Number of chronic conditions. Mean [SD]		6.05 [5.4]	10.26 [5.7]	<0.001	-	12.01 [5.2]	<0.001	-

According to the selected codes, 1352 of the patients (9.5%; 95% CI: 9.0–9.9) suffered a severe infection. The most frequent severity criteria were oxygen therapy (n = 891, 6.2%), and respiratory failure (n = 880, 6.2%). Regarding isolated mortality, 555 patients (3.9%; 95% CI: 3.6–4.2) died during a COVID-19 associated episode, with 368 cases (66.3%) occurring at a hospital.

The relationship between severity / mortality and the demographic and medical variables (age, sex, MM, and number of CC), identified by bivariate statistical analyses, is presented in Table 1. Descriptive statistics of severe COVID-19 infection stratified by confirmed or suspected cases are shown in S2 Table.

### Chronic conditions of the COVID-19 patients

A total of 215 CC were identified, belonging to 20 different families of diseases. The most represented families were *Diseases of the circulatory system* (which contained 33 CC), *Diseases of the genitourinary system* (22 CC), *Mental*, *behavioural and neurodevelopmental disorders* (22 CC), and *Diseases of the musculoskeletal system and connective tissue* (21 CC). The most prevalent CC were *Essential hypertension* (30.3%), *Anxiety and fear-related disorders* (26.62%), *Obesity* (21.8%), and *Disorders of lipid metabolism* (21.3%).

For each age-sex stratum, different sets of CC with prevalence equal to or greater than 2% were identified and are listed in S3 Table.

### Multimorbidity patterns

Fuzzy c-means cluster analysis was performed with a *m* value of 1.1, and *C* values were set to 4 clusters (males of 21–45 years; females of 81–95 years), 5 clusters (males of 81–90 years; females of 21–45, 46–65, and 66–80 years), and 6 clusters (males of 46–65 and 66–80 years).

**Fig 1:**
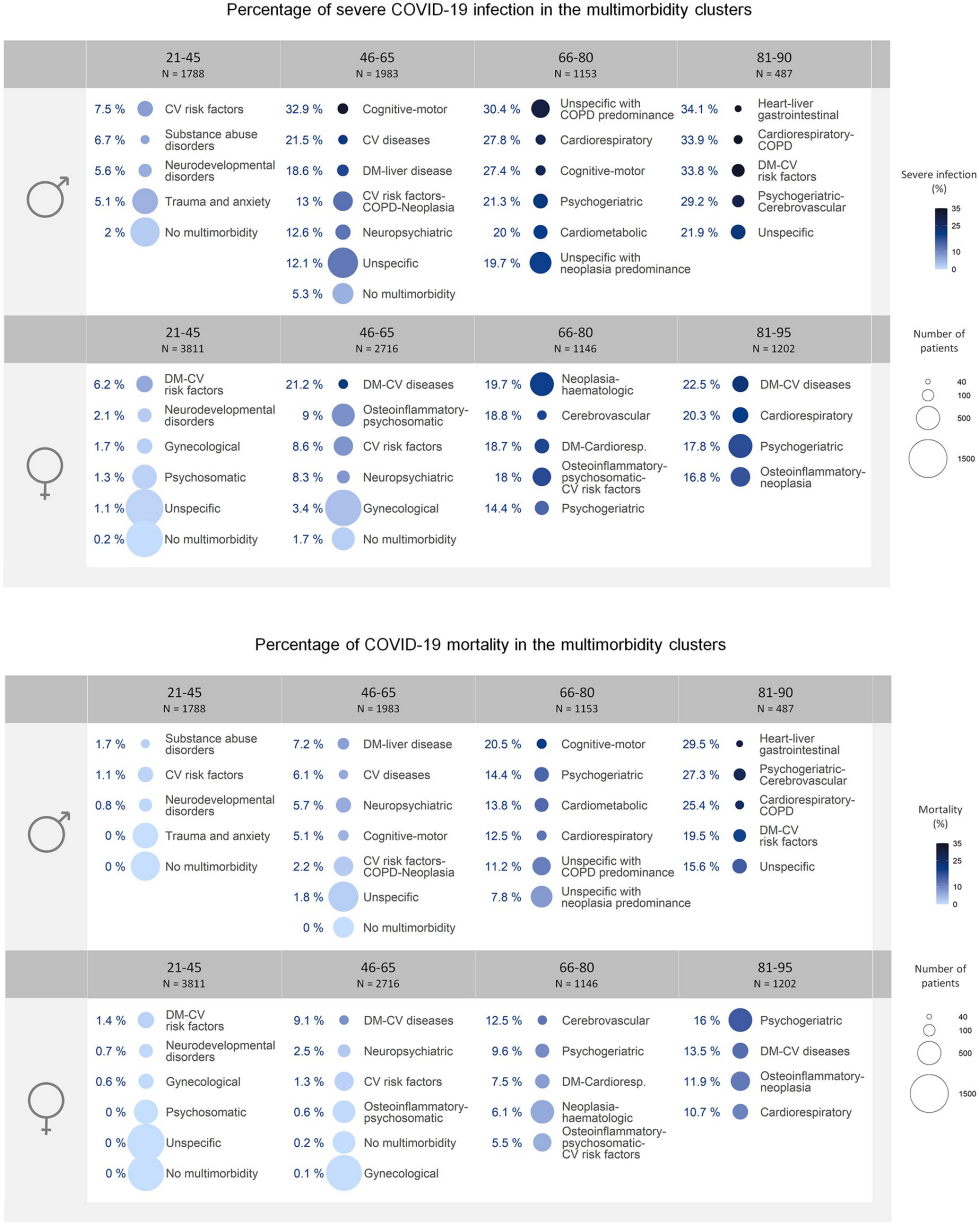
List of multimorbidity clusters per age and sex stratum. The size of the circles represents the population size and the color indicates the percentage of severe COVID-19 cases (upper figure) and mortality (lower figure). COPD = Chronic Obstructive Pulmonary Disease; CV = Cardiovascular.

The resulting MM clusters showed distinct CC distribution in each age-sex stratum. Detailed information on the prevalence, exclusivity, and O/E ratio of each CC per cluster is provided in S4 Table. S2 and S3 Figs illustrate the most representative CC per cluster. The distribution of patients among the different clusters, estimated based on their membership percentages, is summarized in Fig 1 and Table 2.

**Table 2:** Distribution of patients per cluster and infection severity or mortality. COPD = Chronic obstructive pulmonary disease; DM = Diabetes mellitus; OR = Odds ratio; CI = Confidence interval; (*) = not included in the bivariate analysis.

	Sex	Cluster	Total	Severe infection	p-value	OR [95% CI]	Mortality	p-value	OR [95% CI]
21–45	Male	Substance abuse disorders	60	4 (6.7%)	0.001	3.5 [0.9–9.9]	1 (1.7%)	0.052	2.2 [0.1–86.7]
		Neurodevelopmental disorders	126	7 (5.6%)		3.1 [1.2–7.2]	1 (0.8%)		Reference
		Cardiovascular risk factors	186	14 (7.5%)		7.2 [3.5–15.0]	2 (1.1%)		2.1 [0.2–65.6]
		Trauma and anxiety	602	31 (5.1%)		2.2 [1.2–4.3]	0 (0.0%)		-
		No multimorbidity	814	16 (2.0%)		Reference	0 (0.0%)		-
		Total	1788	72 (4.0%)			4 (0.2%)		
	Female	Neurodevelopmental disorders	141	3 (2.1%)	<0.001	9.6 [1.6–56.5]	1 (0.7%)	<0.001	1.2 [0.0–47.6]
		Gynecological disorders	174	3 (1.7%)		7.9 [1.3–46.3]	1 (0.6%)		Reference
		DM and cardiovascular risk factors	209	13 (6.2%)		28.6 [9.0–131.5]	3 (1.4%)		2.3 [0.3–66.5]
		Psychosomatic	532	7 (1.3%)		5.9 [1.6–28.9]	0 (0.0%)		-
		Unspecific	1420	16 (1.1%)		4.8 [1.6–21.5]	0 (0.0%)		-
		No multimorbidity	1335	3 (0.2%)		Reference	0 (0.0%)		-
		Total	3811	45 (1.2%)			5 (0.1%)		
46–65	Male	Cardiovascular diseases	65	14 (21.5%)	<0.001	4.8 [2.2–10.0]	4 (6.1%)	<0.001	3.6 [1.0–10.2]
		Cognitive-motor disorders	79	26 (32.9%)		9.3 [4.9–18.1]	4 (5.1%)		3.0 [0.8–8.5]
		DM—liver diseases	97	18 (18.6%)		3.5 [1.7–7.2]	7 (7.2%)		4.3 [1.6–10.4]
		Neuropsychiatric	174	22 (12.6%)		2.6 [1.4–5.0]	10 (5.7%)		3.6 [1.6–7.8]
		Cardiovascular risk factors—COPD—Neoplasia	316	41 (13.0%)		2.5 [1.4-4-4]	7 (2.2%)		1.1 [0.4–2.7]
		Unspecific	876	106 (12.1%)		2.5 [1.5–4.2]	16 (1.8%)		Reference
		No multimorbidity	376	20 (5.3%)		Reference	0 (0.0%)		-
		Total	1983	247 (12.5%)			48 (2.4%)		
	Female	DM—cardiovascular diseases	66	14 (21.2%)	<0.001	16.3 [6.7–42.7]	6 (9.1%)	<0.001	40.5 [6.5–1060.7]
		Neuropsychiatric	120	10 (8.3%)		5.1 [1.9–13.8]	3 (2.5%)		10.7 [1.2–309.5]
		Cardiovascular risk factors	304	26 (8.6%)		5.3 [2.5–12.8]	4 (1.3%)		5.4 [0.7–149.4]
		Osteoinflammatory—psychosomatic	456	41 (9.0%)		5.2 [2.5–12.2]	3 (0.6%)		2.8 [0.3–80.4]
		Gynecological disorders	1311	44 (3.4%)		1.9 [0.9–4.5]	2 (0.1%)		0.6 [0.1–20.7]
		No multimorbidity	459	8 (1.7%)		Reference	1 (0.2%)		Reference
		Total	2716	143 (5.3%)			19 (0.7%)		
66–80	Male	Cardiorespiratory	72	20 (27.8%)	0.016	1.5 [0.8–2.6]	9 (12.5%)	0.020	1.6 [0.7–3.4]
		Cognitive-motor disorders	73	20 (27.4%)		1.2 [0.6–2.2]	15 (20.5%)		3.1 [1.5–6.0]
		Cardiometabolic	145	29 (20.0%)		1.1 [0.6–1.7]	20 (13.8%)		1.8 [1.0–3.3]
		Psychogeriatric	160	34 (21.3%)		1.1 [0.7–1.7]	23 (14.4%)		1.9 [1.1–3.4]
		Unspecific with COPD predominance	276	84 (30.4%)		1.9 [1.4–2.8]	31 (11.2%)		1.4 [0.8–2.4]
		Unspecific with neoplasia predominance	412	81 (19.7%)		Reference	32 (7.8%)		Reference
		No multimorbidity (*)	15	3 (20.0)		-	1 (6.7%)		-
		Total	1153	271 (23.5%)			131 (11.4%)		
	Female	Cerebrovascular	64	12 (18.8%)	0.738	1.2 [0.5–2.7]	8 (12.5%)	0.215	2.2 [0.8–5.5]
		Psychogeriatric	146	21 (14.4%)		Reference	14 (9.6%)		1.9 [0.9–4.2]
		DM—cardiorespiratory	160	30 (18.8%)		1.3 [0.7–2.5]	12 (7.5%)		1.5 [0.7–3.3]
		Osteoinflammatory—psychosomatic—cardiovascular risk factors	272	49 (18.0%)		1.2 [0.7–2.2]	15 (5.5%)		Reference
		Neoplasia—haematologic	493	97 (19.7%)		1.3 [0.8–2.3]	30 (6.1%)		1.0 [0.6–2.1]
		No multimorbidity (*)	11	0 (0.0%)		-	0 (0.0%)		-
		Total	1146	209 (18.2%)			79 (6.9%)		
81–95	Male	Heart—liver—gastrointestinal diseases	44	15 (34.1%)	0.178	2.2 [1.0–4.7]	13 (29.5%)	0.084	2.8 [1.2–6.1]
		Cardiorespiratory—COPD	59	20 (33.9%)		1.6 [0.8–3.2]	15 (25.4%)		1.7 [0.8–3.5]
		Psychogeriatric/cerebrovascular	106	31 (29.2%)		1.5 [0.9–2.7]	29 (27.3%)		2.3 [1.2–4.2]
		DM—cardiovascular risk factors	118	40 (33.9%)		1.7 [1.0–3.0]	23 (19.5%)		1.3 [0.7–2.4]
		Unspecific	160	35 (21.9%)		Reference	25 (15.6%)		Reference
		No multimorbidity (*)	0	0 (-)		-	0 (-)		-
		Total	487	141 (29.0%)			105 (21.6%)		
	Female	Cardiorespiratory	187	38 (20.3%)	0.379	1.4 [0.9–2.2]	20 (10.7%)	0.198	Reference
		DM—cardiovascular diseases	200	45 (22.5%)		1.4 [0.9–2.2]	27 (13.5%)		1.3 [0.7–2.5]
		Osteoinflammatory—neoplasia	310	52 (16.8%)		Reference	37 (11.9%)		1.0 [0.6–1.8]
		Psychogeriatric	501	89 (17.8%)		1.2 [0.8–1.7]	80 (16.0%)		1.7 [1.1–3.0]
		No multimorbidity (*)	4	0 (0.0%)		-	0 (0.0%)		-
		Total	1202	224 (18.6%)			164 (13.6%)		

Clusters involving DM or CV conditions / risk factors were found in all age-sex groups. *Neurodevelopmental disorders* clusters were present in both groups of 21–45 year-olds, and *Neuropsychiatric* patterns were found in the 46–65 year-olds’ groups. Clusters grouping *Psychogeriatric* diseases were identified in patients of >65 years, along with the *Cardiorespiratory* and *DM—cardiorespiratory* patterns. Some clusters were sex-specific, like *Cognitive-motor disorders* in males (46–65 and 66–80 years), and osteoinflammatory conditions in females, encompassing the clusters *Osteoinflammatory—psychosomatic* (46–65 years), *Osteoinflammatory—psychosomatic—CV risk factors* (66–80 years), and *Osteoinflammatory—neoplasia* (81–95 years).

### Relationship between multimorbidity clusters and severe COVID-19 infection or mortality

The distribution of severe cases and mortality among MM clusters is represented in Fig 1 and detailed in Table 2 along with the *P*-values and odds ratios.

The weighted bivariate analysis showed that clusters of patients in the age ranges of 21–45 and 46–65 displayed *P*≤0.001 in relation to severe infection, and clusters of males in the 66–80 cohort presented *P* = 0.016. Among male patients, the clusters with the highest percentage of severe cases were *CV risk factors* (21–45 years, 7.5%), *Cognitive-motor disorders* (46–65 years, 32.9%), *Unspecific with COPD predominance* (66–80 years, 30.4%), and *Heart—liver—gastrointestinal diseases* (81–90 years, 34.1%). In female patients, the clusters associated with the highest percentages of severity were *DM and CV risk factors* (21–45 years, 6.2%), *DM—CV diseases* (46–65 years, 21.2%), *Neoplasia—haematologic* (66–80 years, 19.7%), and *DM—CV diseases* (81–95 years, 22.5%).

Regarding mortality, clusters of females aged 21–65 and males aged 46–65 obtained *P*<0.001, and males aged 66–80 presented *P* = 0.02. The clusters with the highest percentage of mortality among males were *Substance abuse disorders* (21–45 years, 1.7%), *DM—liver diseases* (46–65 years, 7.2%), *Cognitive-motor disorders* (66–80 years, 20.5%), and *Heart—liver—gastrointestinal diseases* (81–90 years, 29.5%). Clusters with the highest percentage of mortality in females were *DM and CV risk factors* (21–45 years, 1.4%), *DM—CV diseases* (46–65 years, 9.1%), *Cerebrovascular diseases* (66–80 years, 12.5%), and *Psychogeriatric* (81–95 years, 16.0%).

## Discussion

The present study assessed the MM patterns associated to the population affected by the first wave of COVID-19 in a Spanish area. The results revealed well-defined MM clusters taking into account age and sex, some of which showed a high severity of COVID-19 infection and mortality, with a stronger relationship in younger age groups.

First of all, defining the study cohort was a vital aspect in order to establish reliable MM profiles. Many studies from the first wave of the pandemic focused only on PCR positive individuals [1, 3, 9, 38]; nevertheless, the critical situation at that time resulted in a small fraction of patients undergoing confirmatory tests in the studied geographic area. The test positivity rate in Catalonia during the selected period peaked at 43.5% and did not drop below the World Health Organization recommendation of 5% until mid-May, thereby showing the shortage of testing resources [39, 40]. This situation particularly affected older individuals, who suffered the highest mortality rates in nursing homes without testing nor hospital admission [41]. Therefore, excluding these individuals from the cohort could introduce biases in the definition of multimorbidity patterns.

Taking the whole situation into account, the inclusion of suspected cases in the study cohort allowed for a more accurate approximation of the actual affected population. Although this criterion may have resulted in some possible false positives, it helped avoid potential biases associated with factors like age, access to medical testing or socioeconomic situation.

Using fuzzy c-means cluster analysis allowed for the simultaneous classification of patients into different clusters with a certain probability. This method is robust for pattern recognition in situations where clusters tend to overlap [42], such as in individuals with a high prevalence of co-existing conditions. It should be taken into account that the number of clusters *C* depends on the researchers’ subjective election, which could affect the reproducibility of the study. Nevertheless, a Delphi-like method was carried out in order to enhance the robustness to the cluster selection, taking advantage of the multidisciplinarity of the research team, which includes members with technical or clinical expertise [43].

In addition, another key methodological aspect of this work was the stratification of the cohort. Given that the accumulation of CC is more frequent at older ages [17, 44], and that males and females have differential tendencies to suffer certain pathologies [45], it was necessary to consider both age and sex perspectives. In fact, previous studies on COVID-19 data from different countries already revealed that men have a higher risk of admission to intensive treatment units (from 14.4% to 44.9%) [1] or adverse outcome (OR: 2.05, 95% CI: 1.39–3.04) [2], which was supported by our bivariate analysis of demographic data. Indeed, several sex-related differences were observed in the MM clusters: patterns related with psychosomatic conditions or osteoinflammatory diseases were more frequent in females, while cognitive-motor, digestive and respiratory disease patterns were more associated to males, consistent with previous findings [23, 46, 47].

Regarding age-related differences, previous works based on the fuzzy c-means algorithm already identified certain MM clusters in cohorts of older patients (≥60 years) that were similar to some patterns observed in the 66–80 and 81–95 age groups in the present study. For example, Vetrano et al identified clusters of cardiometabolic diseases, respiratory diseases, and cognitive / sensory impairment [28], and Akugizibwe et al found clusters of psychiatric conditions and sensory impairments/cancer [27]. Regarding the younger cohorts, a previous stratified study by Prados-Torres et al found some similar clusters by factor analysis, like psychiatric-substance abuse (males of 15–45 years), or cardio-metabolic (all strata) [48].

On the basis of these clusters, the results of the bivariate analyses suggest that certain MM patterns could be associated to higher COVID-19 infection severity or mortality rates in young-middle ages. The fact that this relationship was not strong in older patients may be explained by the inherent risk of old age itself [49, 50], as indicated by the bivariate analysis of demographic parameters. Additionally, other factors could also explain this mild association in older individuals, such as the simple accumulation of morbidities [17, 51, 52], difficulties in accessing healthcare services during the first wave of the pandemic [53], or the adequacy of therapeutic effort policy in age-related cases [54]. Furthermore, geriatric syndromes [55], which are barely coded in medical records, represent an important factor of vulnerability [25]. For example, frailty and disability are commonly detected in older patients and have been described as potential risk factors of critical COVID-19 infection [56, 57]. Nevertheless, in spite of the lack of strong statistical association, several MM clusters of patients in the 66–80 and 81–95 age ranges showed elevated infection severity and mortality rates compared to other clusters.

Therefore, the high percentages of infection severity / mortality displayed by certain MM clusters suggest that these patients may be more vulnerable to poor prognosis caused by COVID-19 infection. Previous pre-pandemic studies already showed some similar MM patterns associated with other adverse outcomes. For example, clusters of psychiatric disorders and CV diseases have been linked to a higher risk of unplanned hospitalization [27], while cognitive / sensory, complex cardiometabolic, respiratory, and age-associated chronic clusters showed increased risk of mortality [28, 58]. Taking into account other COVID-19 studies focusing on individual diseases, several CC that showed high prevalence and O/E ratio in the vulnerable clusters of our study were already identified as risk factors of adverse outcomes, such as COPD [4, 5, 8], liver disease, substance consumption [7], neoplasia [5, 33], and CV pathologies [31, 33]. Nevertheless, the present results indicate that beyond the occurrence of isolated pathologies, the accumulation of CC may represent an additional dimension to consider when facing patient-centered risk evaluations, as suggested by other authors [12].

In any case, the described results should always be interpreted considering the limitations of the study. Although the inclusion criteria took into account the characteristics of the first wave of the pandemic, there are inherent limitations related to this period (concerning data collection, codification and testing). Additionally, it should be considered that Big Data analyses imply some disadvantages, such as difficulties in ensuring data quality and homogeneity [59]. On the other hand, the study data corresponds to a specific region, which ensures a certain uniformity in the characteristics of the population, as well as their clinical management, protocols and codification, mitigating the potential heterogeneity of Big Data. Lastly, it should be noted that the occurrence of COVID-19 severe infection or mortality, or even their relationship with MM clusters, might be influenced by additional factors that were not included in the analyses, such as healthcare-associated features, socioeconomic status, dependency or frailty.

Therefore, these results provide insights into the relationships between MM clusters and severity or mortality, but do not provide a risk prediction. Our methodological approach allowed to explore if a comprehensive set of conditions forming multimorbidity clusters was related to the risk of COVID-19 infection severity or mortality. This approach may offer a complementary picture to the findings of possible further studies with multivariate analyses.

## Conclusions

In summary, the present study provided a complete stratified profiling of MM clusters in COVID-19 patients from the first wave of the pandemic, revealing that males, older individuals and patients with certain MM patterns displayed higher probabilities of suffering severe COVID-19 infection or mortality. These findings may contribute to enhance the current knowledge about MM definition as well as to identify the most vulnerable population. This might especially benefit the preventive care of high-risk patients or those with limited access to medical treatment, and might help optimize healthcare policies and future strategies.

## Supporting information

S1 Table List of codes for COVID-19 registration, primary care mortality and severe infection. ICD-10-CM = International Classification of Diseases, 10th edition, Clinical Modification. ICD-10-PCS = International Classification of Diseases, 10th edition, Procedure Coding System.
(PDF)

S2 Table Number of patients and prevalence of severe COVID-19 infection according to the type of case (confirmed or suspected). (PDF)

S3 Table Chronic conditions with >2% prevalence per age and sex stratum. (XLSX)

S4 Table Prevalence (%), Observed/Expected (O/E) ratio, and exclusivity (%) of the chronic conditions per multimorbidity clusters. (XLSX)

S1 Fig Source and distribution of the analysed data. The included patients were submitted to an anonymization process. A patient Id code was assigned to each individual, which was maintained through all the different datasets in order to enable data compilation.
(PDF)

S2 Fig Representation of the Observed/Expected ratio (O/E) and exclusivity (%) of the chronic conditions composing the multimorbidity clusters of male patients. O/E >1 and exclusivity > 1/number of clusters are displayed.
(PDF)

S3 Fig Representation of the Observed/Expected ratio (O/E) and exclusivity (%) of the chronic conditions composing the multimorbidity clusters of female patients. O/E >1 and exclusivity > 1/number of clusters are displayed.
(PDF)

## Abbreviations

AQuAS: Agency for Health Quality and Assessment of Catalonia
CC: Chronic condition
COPD: Chronic obstructive pulmonary disease
CV: Cardiovascular
DM: Diabetes mellitus
HC3: Shared Clinical History of Catalonia
ICD-10-CM: International Classification of Diseases, 10th edition, Clinical Modification
ICD-10-PCS: International Classification of Diseases, 10th edition, Procedure Coding System
MM: Multimorbidity
O/E: Observed/Expected
PADRIS: Public Data Analysis for Health Research and Innovation Program
SUVEC: Catalan Epidemiological Surveillance Emergency Service
