# Supplementary material for: Multimorbidity patterns in COVID-19 patients and their relationship with infection severity: MRisk-COVID study
Source: PLoS One. 2023 Aug 31;18(8):e0290969. doi: 10.1371/journal.pone.0290969 (PMC10470964; doi:10.1371/journal.pone.0290969)
Supplement: S1 Table — ICD-10-CM = International Classification of Diseases, 10th edition, Clinical Modification. ICD-10-PCS = International Classification of Diseases, 10th edition, Procedure Coding System. (PDF) [file pone.0290969.s001.pdf]

Table S1. List of codes for COVID-19 registration, primary care mortality and severe infection.

ICD-10-CM = International Classification of Diseases, 10th edition, Clinical Modification

ICD-10-PCS = International Classification of Diseases, 10th edition, Procedure Coding System

**CODES FOR COVID-19 REGISTRATION**		
**ICD-10-CM**	**Description**	
B342	Coronavirus infection, unspecified	
B9721	SARS-associated coronavirus as the cause of diseases classified elsewhere	
B9729	Other coronavirus as the cause of diseases classified elsewhere	
J1281	Pneumonia due to SARS-associated coronavirus	
J1289	Other viral pneumonia	
U071	COVID-19	
Z20828	Contact with and (suspected) exposure to other viral communicable diseases	
**CODES FOR MORTALITY (PRIMARY CARE)**		
**ICD-10-CM**	**Description**	**Category**
R99	Ill-defined and unknown cause of mortality	Mortality
**CODES FOR SEVERE INFECTION: DIAGNOSES**		
**ICD-10-CM**	**Description**	**Category**
J80	Acute respiratory distress syndrome	ARDS
R6511	Systemic inflammatory response syndrome (SIRS) of non-infectious origin with acute organ dysfunction	Inflammatory response
R6520	Severe sepsis without septic shock	Inflammatory response
D65	Disseminated intravascular coagulation [defibrination syndrome]	Organ failure
E271	Primary adrenocortical insufficiency	Organ failure
I2609	Other pulmonary embolism with acute cor pulmonale	Organ failure
I2699	Other pulmonary embolism without acute cor pulmonale	Organ failure
I501	Left ventricular failure, unspecified	Organ failure
I5020	Unspecified systolic (congestive) heart failure	Organ failure
I5021	Acute systolic (congestive) heart failure	Organ failure
I5030	Unspecified diastolic (congestive) heart failure	Organ failure
I5031	Acute diastolic (congestive) heart failure	Organ failure
I5043	Acute on chronic combined systolic (congestive) and diastolic (congestive) heart failure	Organ failure
I50810	Right heart failure, unspecified	Organ failure
I50811	Acute right heart failure	Organ failure
I50813	Acute on chronic right heart failure	Organ failure
I5082	Biventricular heart failure	Organ failure
I5083	High output heart failure	Organ failure
I5089	Other heart failure	Organ failure
I509	Heart failure, unspecified	Organ failure
K7200	Acute and subacute hepatic failure without coma	Organ failure
K7201	Acute and subacute hepatic failure with coma	Organ failure
K7290	Hepatic failure, unspecified without coma	Organ failure
K7291	Hepatic failure, unspecified with coma	Organ failure
N170	Acute kidney failure with tubular necrosis	Organ failure
N171	Acute kidney failure with acute cortical necrosis	Organ failure
N172	Acute kidney failure with medullary necrosis	Organ failure
N178	Other acute kidney failure	Organ failure
N179	Acute kidney failure, unspecified	Organ failure
N19	Unspecified kidney failure	Organ failure
N990	Postprocedural (acute) (chronic) kidney failure	Organ failure
J9600	Acute respiratory failure, unspecified whether with hypoxia or hypercapnia	Respiratory failure
J9601	Acute respiratory failure with hypoxia	Respiratory failure
J9602	Acute respiratory failure with hypercapnia	Respiratory failure
J9620	Acute and chronic respiratory failure, unspecified whether with hypoxia or hypercapnia	Respiratory failure

J9621	Acute and chronic respiratory failure with hypoxia	Respiratory failure
J9622	Acute and chronic respiratory failure with hypercapnia	Respiratory failure
J9690	Respiratory failure, unspecified, unspecified whether with hypoxia or hypercapnia	Respiratory failure
J9691	Respiratory failure, unspecified with hypoxia	Respiratory failure
J9692	Respiratory failure, unspecified with hypercapnia	Respiratory failure
R6521	Severe sepsis with septic shock	Septic shock
**CODES FOR SERIOUS INFECTION: PROCEDURES**		
**ICD-10-PCS**	**Description**	**Category**
5A1935Z	Respiratory Ventilation, Less than 24 Consecutive Hours	Invasive mechanical ventilation
5A1945Z	Respiratory Ventilation, 24-96 Consecutive Hours	Invasive mechanical ventilation
5A1955Z	Respiratory Ventilation, Greater than 96 Consecutive Hours	Invasive mechanical ventilation
5A09357	Assistance with Respiratory Ventilation, Less than 24 Consecutive Hours, Continuous Positive Airway Pressure	Noninvasive mechanical ventilation
5A09358	Assistance with Respiratory Ventilation, Less than 24 Consecutive Hours, Intermittent Positive Airway Pressure	Noninvasive mechanical ventilation
5A09359	Assistance with Respiratory Ventilation, Less than 24 Consecutive Hours, Continuous Negative Airway Pressure	Noninvasive mechanical ventilation
5A0935B	Assistance with Respiratory Ventilation, Less than 24 Consecutive Hours, Intermittent Negative Airway Pressure	Noninvasive mechanical ventilation
5A0935Z	Assistance with Respiratory Ventilation, Less than 24 Consecutive Hours	Noninvasive mechanical ventilation
5A09457	Assistance with Respiratory Ventilation, 24-96 Consecutive Hours, Continuous Positive Airway Pressure	Noninvasive mechanical ventilation
5A09458	Assistance with Respiratory Ventilation, 24-96 Consecutive Hours, Intermittent Positive Airway Pressure	Noninvasive mechanical ventilation
5A09459	Assistance with Respiratory Ventilation, 24-96 Consecutive Hours, Continuous Negative Airway Pressure	Noninvasive mechanical ventilation
5A0945B	Assistance with Respiratory Ventilation, 24-96 Consecutive Hours, Intermittent Negative Airway Pressure	Noninvasive mechanical ventilation
5A0945Z	Assistance with Respiratory Ventilation, 24-96 Consecutive Hours	Noninvasive mechanical ventilation
5A09557	Assistance with Respiratory Ventilation, Greater than 96 Consecutive Hours, Continuous Positive Airway Pressure	Noninvasive mechanical ventilation
5A09558	Assistance with Respiratory Ventilation, Greater than 96 Consecutive Hours, Intermittent Positive Airway Pressure	Noninvasive mechanical ventilation
5A09559	Assistance with Respiratory Ventilation, Greater than 96 Consecutive Hours, Continuous Negative Airway Pressure	Noninvasive mechanical ventilation
5A0955B	Assistance with Respiratory Ventilation, Greater than 96 Consecutive Hours, Intermittent Negative Airway Pressure	Noninvasive mechanical ventilation
5A0955Z	Assistance with Respiratory Ventilation, Greater than 96 Consecutive Hours	Noninvasive mechanical ventilation
3E0F7GC	Introduction of Other Therapeutic Substance into Respiratory Tract, Via Natural or Artificial Opening	Oxygen therapy
3E0F7SF	Introduction of Other Gas into Respiratory Tract, Via Natural or Artificial Opening	Oxygen therapy
5A05121	Extracorporeal Hyperbaric Oxygenation, Intermittent	Oxygen therapy
5A0512C	Extracorporeal Supersaturated Oxygenation, Intermittent	Oxygen therapy
5A05221	Extracorporeal Hyperbaric Oxygenation, Continuous	Oxygen therapy
5A0522C	Extracorporeal Supersaturated Oxygenation, Continuous	Oxygen therapy
5A09357	Assistance with Respiratory Ventilation, Less than 24 Consecutive Hours, Continuous Positive Airway Pressure	Oxygen therapy
5A09457	Assistance with Respiratory Ventilation, 24-96 Consecutive Hours, Continuous Positive Airway Pressure	Oxygen therapy
5A09557	Assistance with Respiratory Ventilation, Greater than 96 Consecutive Hours, Continuous Positive Airway Pressure	Oxygen therapy
