# Supplementary material for: Multimorbidity patterns in COVID-19 patients and their relationship with infection severity: MRisk-COVID study
Source: PLoS One. 2023 Aug 31;18(8):e0290969. doi: 10.1371/journal.pone.0290969 (PMC10470964; doi:10.1371/journal.pone.0290969)
Supplement: S2 Table — (PDF) [file pone.0290969.s002.pdf]

	Severe COVID-19 infection, N (%)				
	Total	21-45	46-65	66-80	81+
Confirmed	930 (29.6)	97 (11.0)	315 (29.9)	357 (53.8)	161 (29.5)
Suspected	184 (1.7)	16 (0.3)	57 (1.6)	61 (3.7)	50 (4.4)
Total	1114 (7.8)	113 (2.0)	372 (7.9)	418 (18.2)	211 (12.5)

Table S2. Number of patients and prevalence of severe COVID-19 infection according to the type of case (confirmed or suspected).
