# Supplementary material for: Multimorbidity patterns in COVID-19 patients and their relationship with infection severity: MRisk-COVID study
Source: PLoS One. 2023 Aug 31;18(8):e0290969. doi: 10.1371/journal.pone.0290969 (PMC10470964; doi:10.1371/journal.pone.0290969)

Additional file 2.

Figure S1: Source and distribution of the analysed data. The included patients were submitted to an anonymization process. A patient ID code was assigned to each individual, which was maintained through all the different datasets in order to enable data compilation.

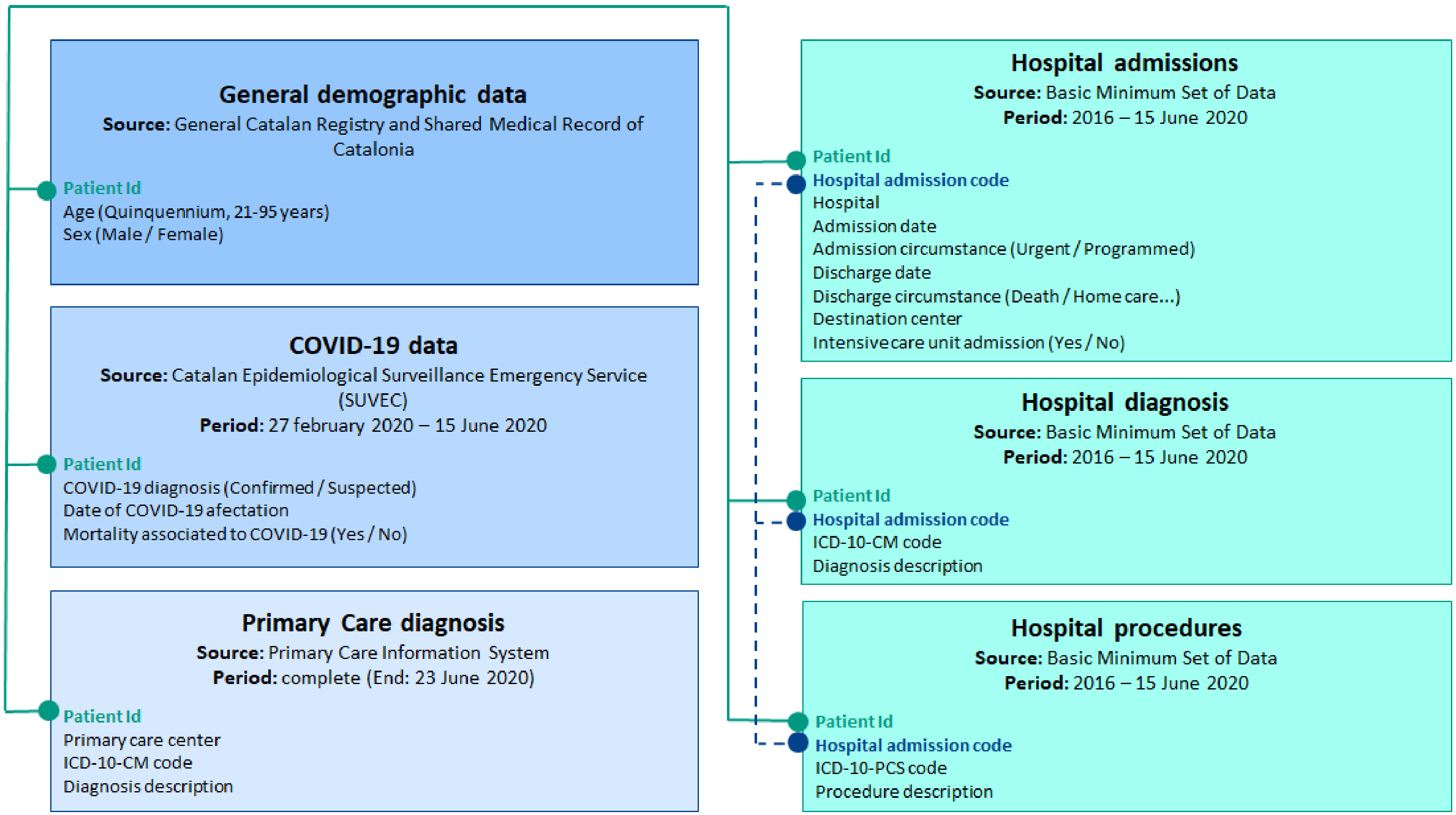

Supplement: S1 Fig — The included patients were submitted to an anonymization process. A patient Id code was assigned to each individual, which was maintained through all the different datasets in order to enable data compilation. (PDF) [file pone.0290969.s005.pdf]
