# Supplementary material for: Multimorbidity patterns in COVID-19 patients and their relationship with infection severity: MRisk-COVID study
Source: PLoS One. 2023 Aug 31;18(8):e0290969. doi: 10.1371/journal.pone.0290969 (PMC10470964; doi:10.1371/journal.pone.0290969)
Supplement: S3 Fig — O/E >1 and exclusivity > 1/number of clusters are displayed. (PDF) [file pone.0290969.s007.pdf]

Additional file 6.

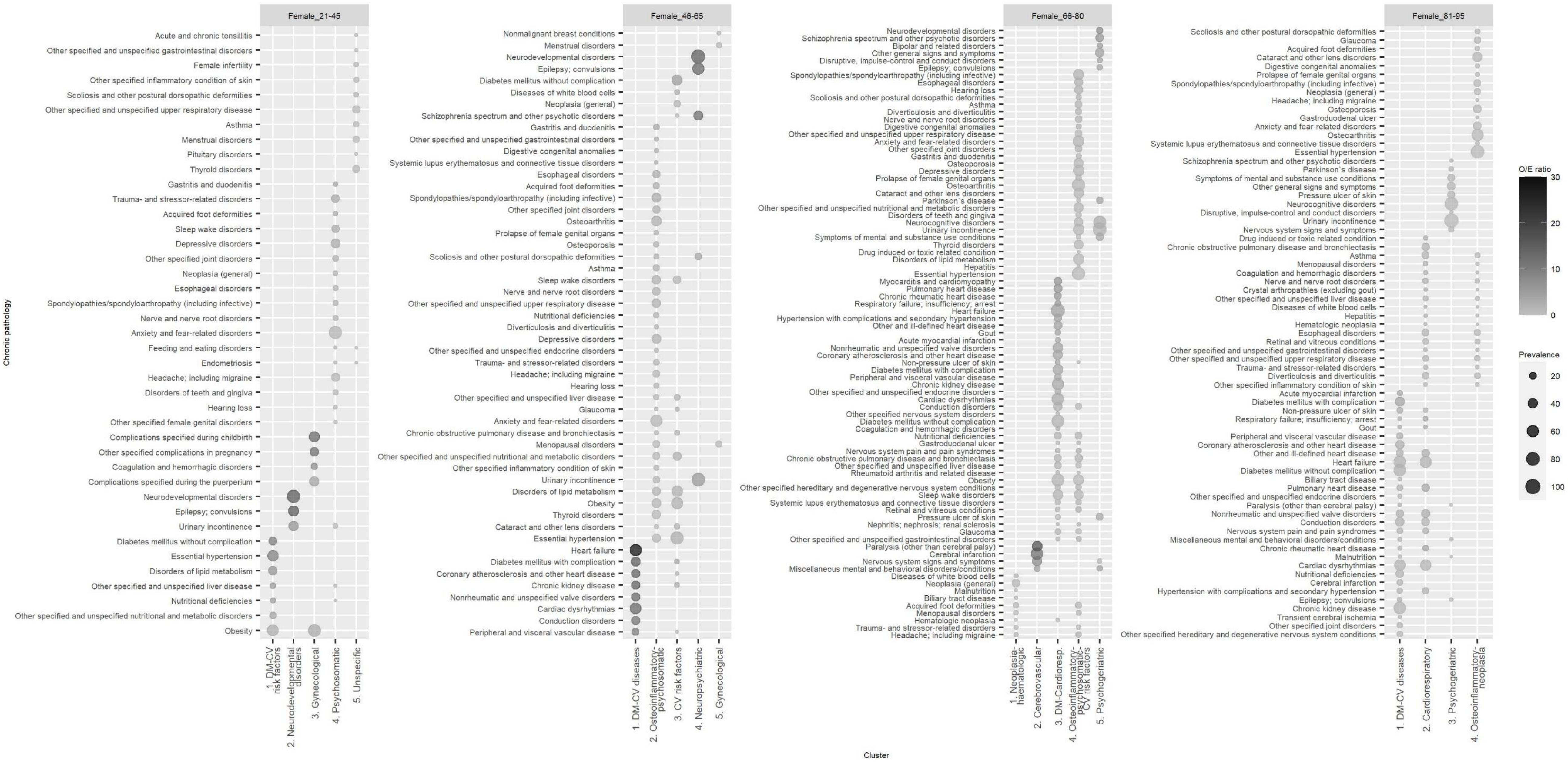

Figure S3: Representation of the Observed/Expected ratio (O/E) and exclusivity (%) of the chronic conditions composing the multimorbidity clusters of female patients. O/E >1 and exclusivity > 1/number of clusters are displayed.
